# Supplementary material for: FDCSP Is an Immune-Associated Prognostic Biomarker in HPV-Positive Head and Neck Squamous Carcinoma
Source: Biomolecules. 2022 Oct 12;12(10):1458. doi: 10.3390/biom12101458 (PMC9599724; doi:10.3390/biom12101458)
Supplement: Supplementary file 1 [file biomolecules-12-01458-s001.zip › biomolecules-1927813-supplmentary.pdf]

Supplementary Materials

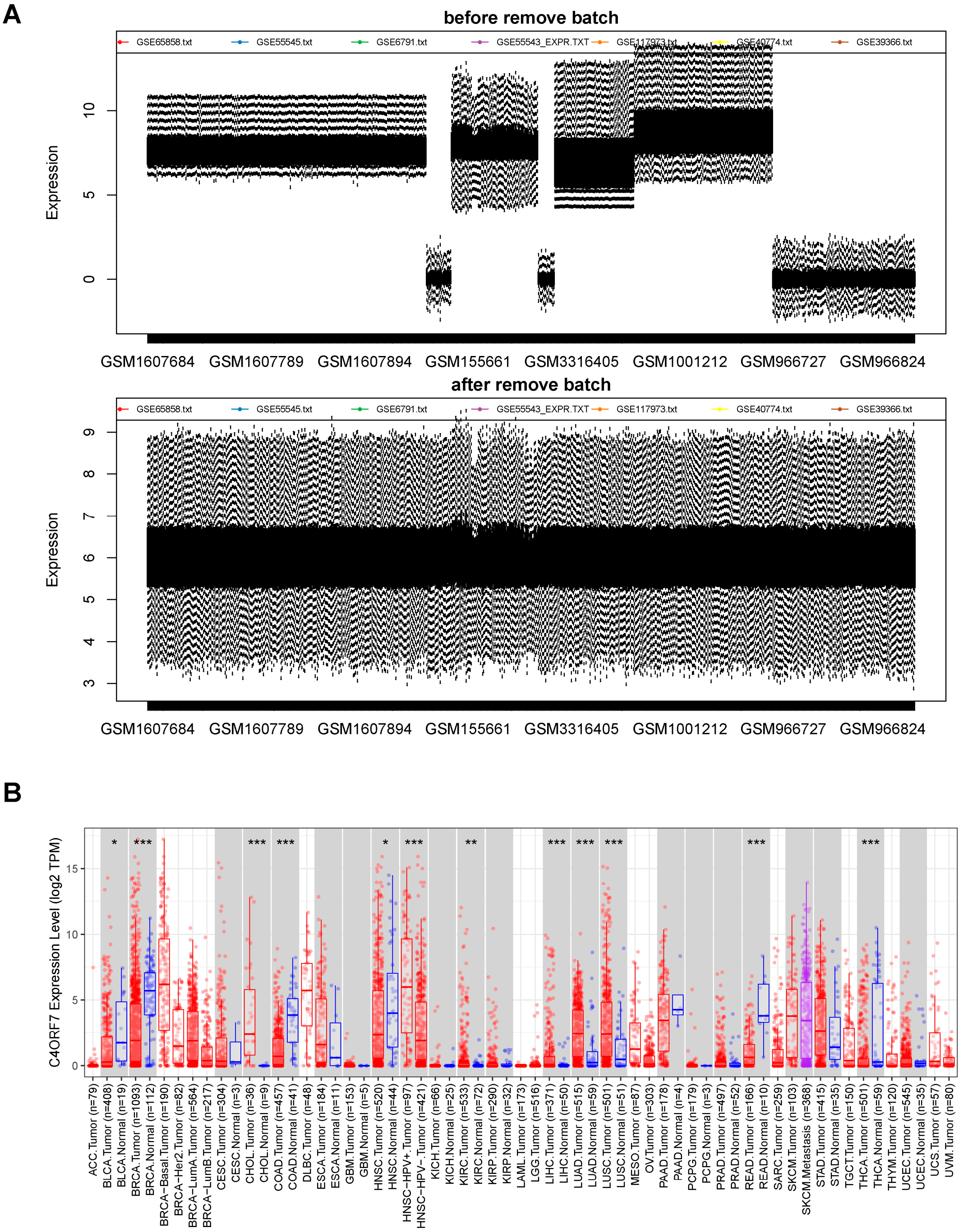

Figure S1. FDCSP expression and GEO cohorts merged.

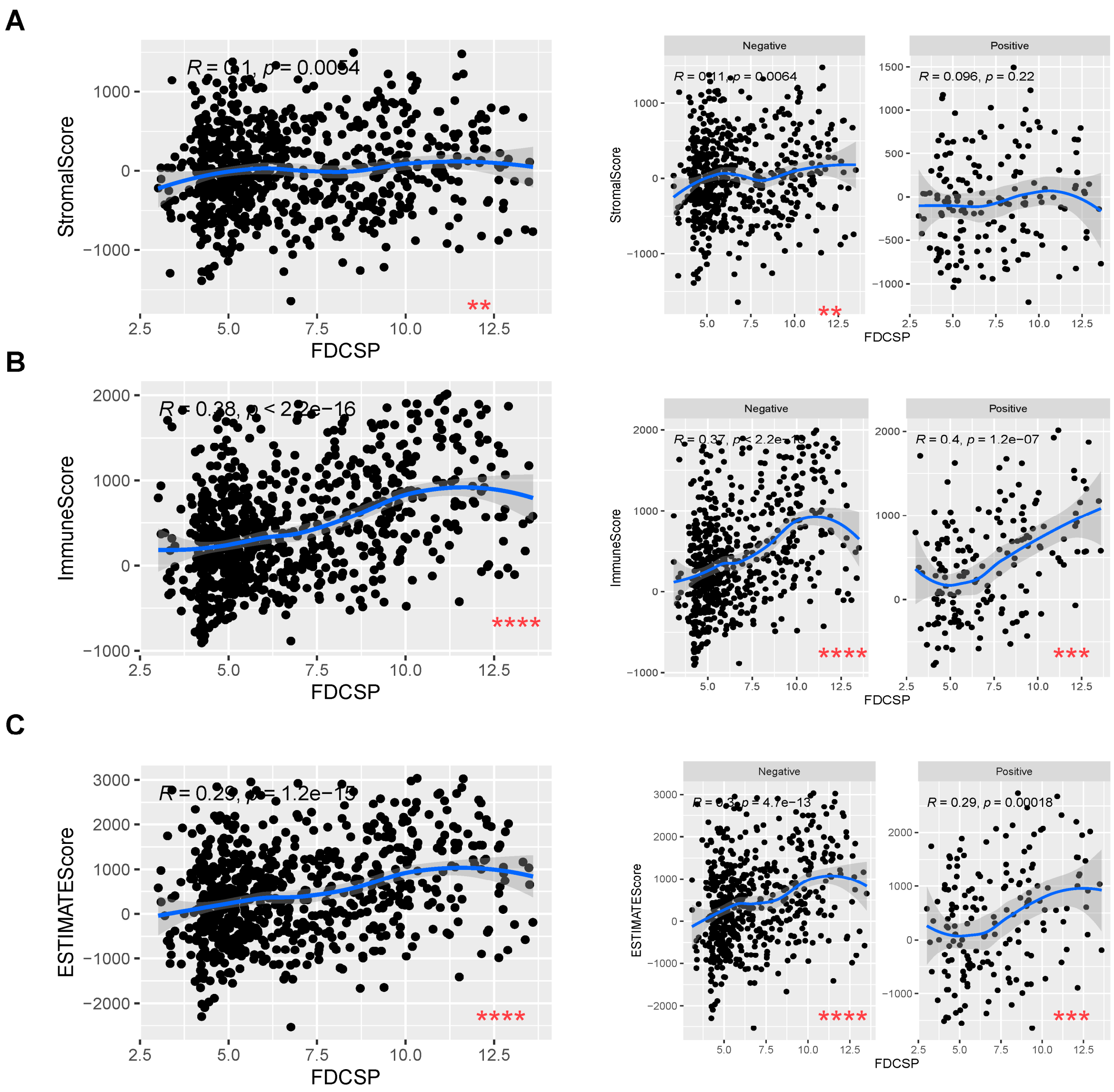

Figure S2. Immune infiltration score associated with FDCSP in HNSC (GEO cohorts).

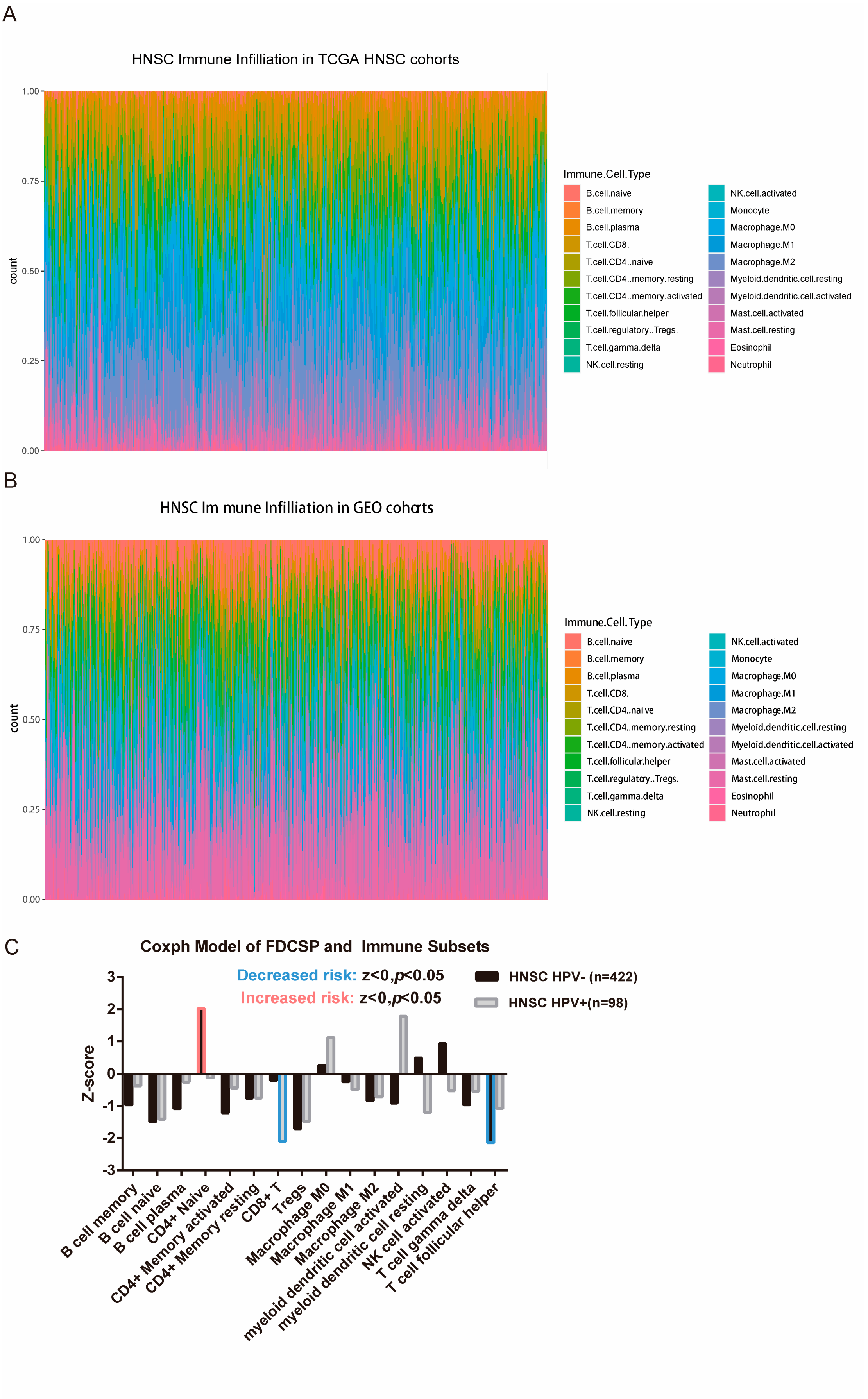

Figure S3. HNSC immune infiltration in TCGA and GEO cohorts.

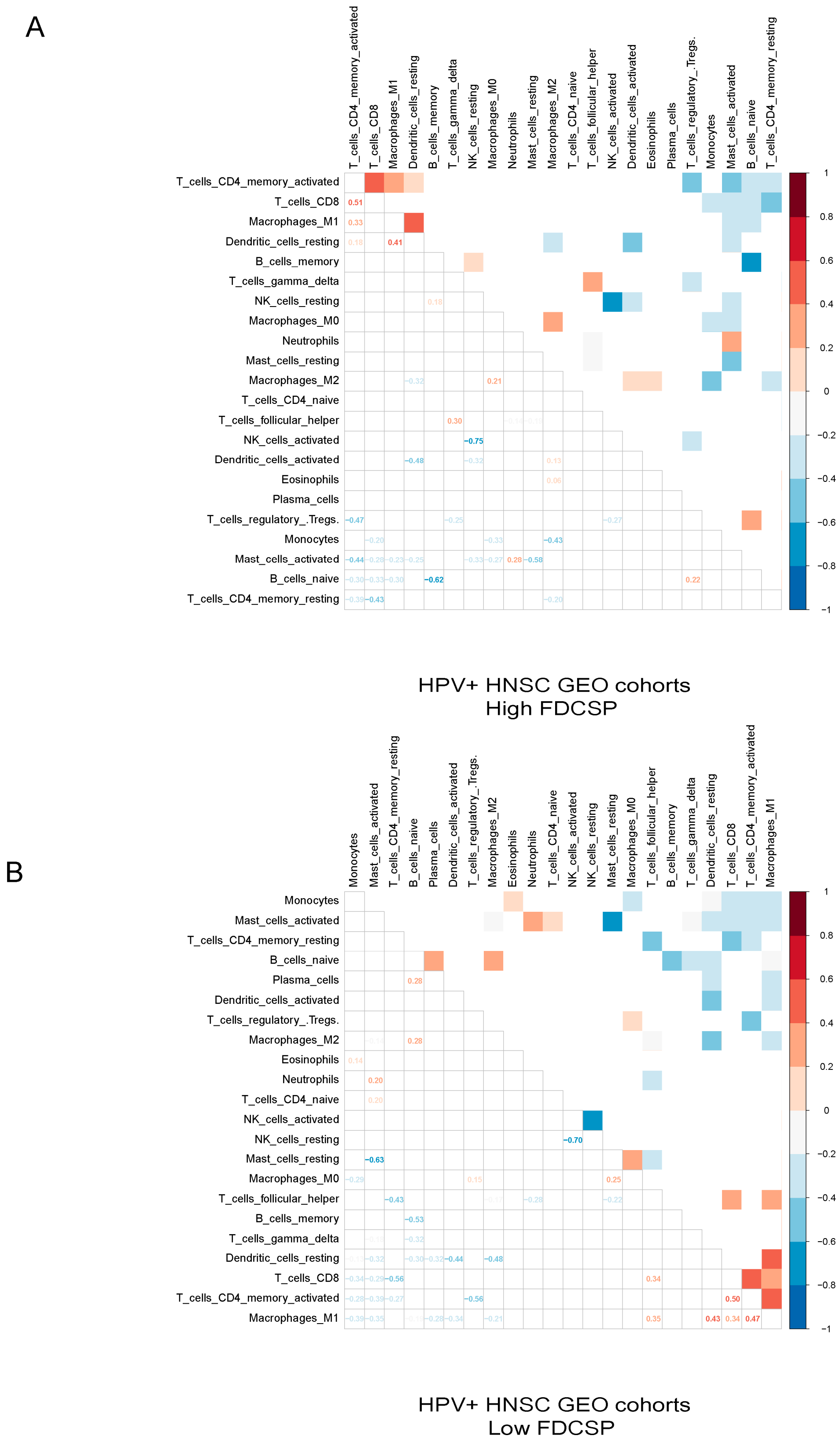

Figure S4. Immune cell correlation in the high/low FDCSP group of HPV+ HNSC (GEO cohorts).

A

B

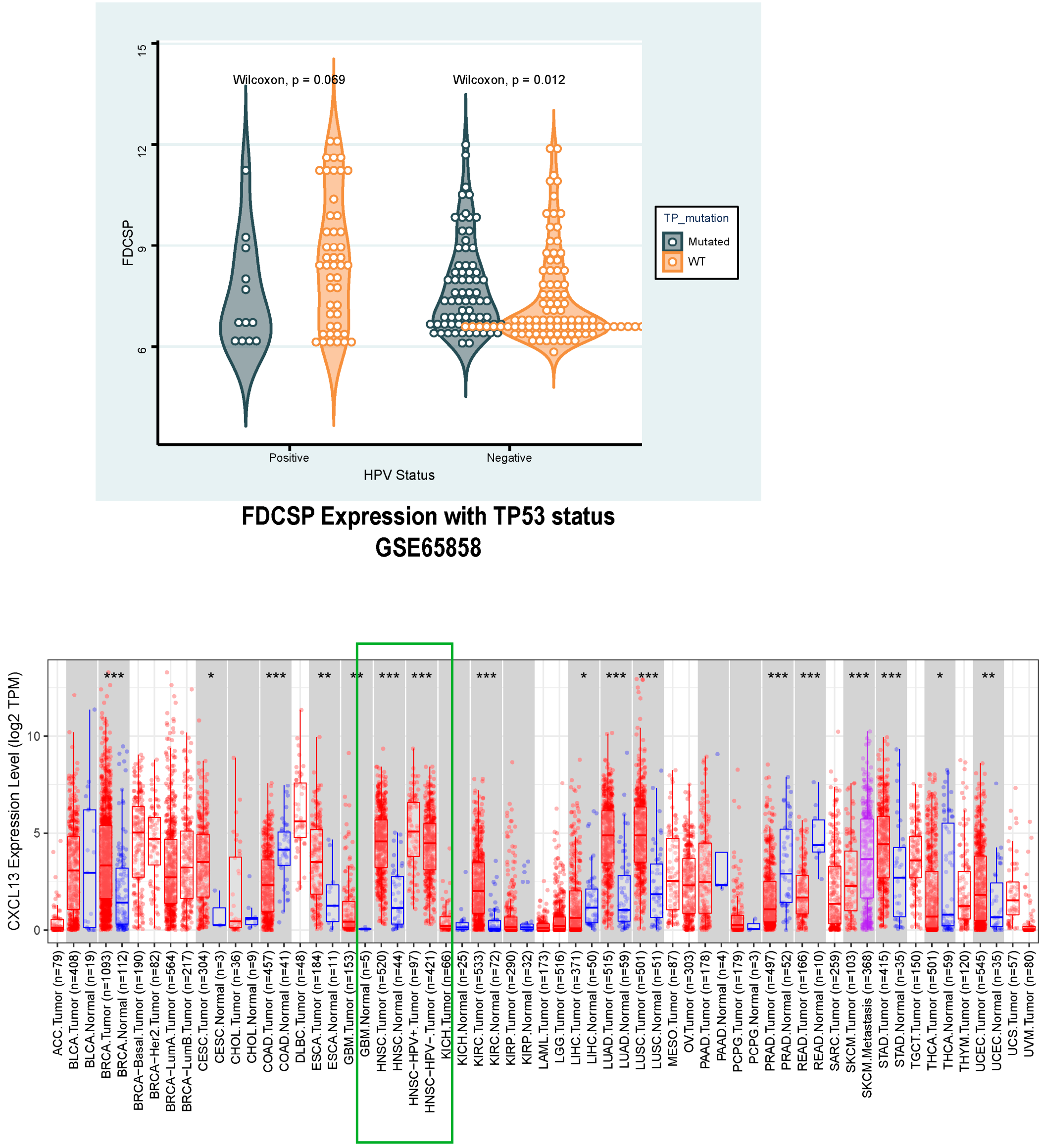

Figure S5. Expression of FDCSP in WT/Mutated TP53 in HPV+/− HNSC (GSE65858 cohort) and the expression of CXCL13 in HNSCs (TCGA, TIMER2.0).
